# Incidental Detection of Situs Inversus Totalis Following Identification of an Unexpected Left-Sided Brachiocephalic Trunk on Carotid Duplex Ultrasound: A Case Report

**DOI:** 10.7759/cureus.105803

**Published:** 2026-03-24

**Authors:** Elias Nabhan, Rida Khouri, Edouard Naoum Nehme, Alexandrina-Paula Vana, Nabil Poulos

**Affiliations:** 1 Cardiology, Centre Hospitalier Josephine Baker, Gonesse, FRA

**Keywords:** brachiocephalic trunk, carotid duplex ultrasound, computed tomography angiography, situs inversus totalis, vascular anomaly

## Abstract

Situs inversus totalis (SIT) is a challenging diagnosis, as failure to recognize the mirrored anatomy can result in complications during surgery, medical procedures, or emergency care. We present the case of a 60-year-old woman with a medical history of dyslipidemia who was referred for a carotid and vertebral artery duplex ultrasound ordered by the referring physician to evaluate subclinical atherosclerosis. The ultrasound unexpectedly showed that the brachiocephalic trunk was not identified on the right side, and a left-sided innominate artery was present, representing an unusual branching configuration. A subsequent contrast-enhanced computed tomography angiography (CTA) of the thorax and abdomen was performed, which revealed a complete situs inversus with mirror-image anatomy of the great vessels and thoracoabdominal viscera. The patient remained completely asymptomatic with no clinical consequences. This case highlights the importance of recognizing atypical vascular anatomy during routine imaging. The observation of an absent right brachiocephalic trunk on a standard Doppler study served as an unusual clue leading to the recognition of mirror-image anatomy.

## Introduction

Situs inversus totalis (SIT) is a congenital disorder that occurs rarely, with an estimated prevalence of one per 5,000 to one per 20,000 live births [1]. It is associated with the total inversion of all the asymmetrical organs in both thoracic and abdominal cavities. Left-right asymmetry is established during early embryogenesis through nodal ciliary motion. Disruption of this motion can lead to laterality disorders, including SIT, characterized by a complete mirror-image configuration of thoracic and abdominal organs [2].

In most cases, SIT is asymptomatic and discovered incidentally during imaging performed for unrelated indications [3,4]. The vascular anatomy, including the aortic arch and its branches, typically demonstrates a mirror-image configuration rather than true anatomical abnormality [5].

We present a case in which a carotid duplex ultrasound, performed to evaluate subclinical atherosclerosis, unexpectedly revealed the absence of the right-sided brachiocephalic trunk. This isolated finding was the key clue that led to the diagnosis of complete SIT in an otherwise healthy 60-year-old woman.

## Case presentation

A 60-year-old woman with a known history of dyslipidemia treated with lifestyle modification was referred to the cardiovascular medicine department for a carotid and vertebral artery duplex ultrasound ordered by the referring physician to evaluate subclinical atherosclerosis. The patient was completely asymptomatic and did not complain of any neurological dysfunction, including dizziness, syncope, transient ischemic attacks, or visual impairments. She did not have any major history of surgery and was not on any regular medication.

**Figure 1:**
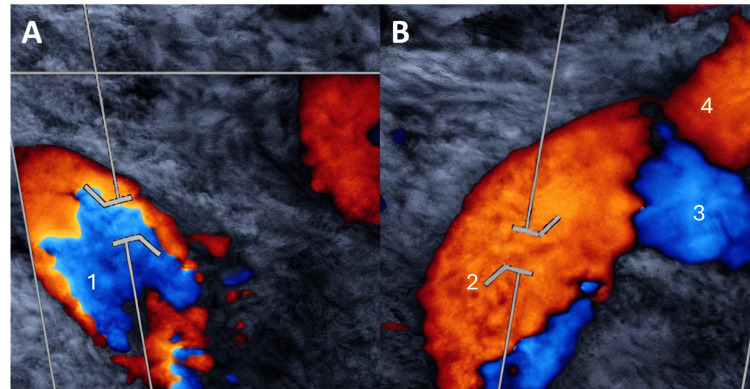
Carotid duplex ultrasound. (A) Color Doppler ultrasound image of the right supraclavicular window demonstrating the right subclavian artery (1). (B) Color Doppler ultrasound image showing the division of the left brachiocephalic trunk (2) into the left subclavian artery (3) and the left common carotid artery (4).

A carotid duplex ultrasound was done with a high-frequency linear array transducer. Primary evaluation of the right supraclavicular fossa, the typical anatomical site for visualizing the brachiocephalic trunk, demonstrated the absence of the right brachiocephalic trunk. Despite thorough scanning, the vessel could not be identified on the right side. Further exploration of the left supraclavicular region revealed an atypical origin of the great vessels. A brachiocephalic trunk was identified arising from the aortic arch on the left side, which then divided into the left common carotid artery and the left subclavian artery. The right common carotid and right subclavian arteries were seen to arise separately from a right-sided aortic arch, creating a perfect mirror-image of the standard configuration seen in SIT (Figure 1). Aside from this anatomical variant, the intima-media thickness of the visualized carotid arteries was minimally increased, as expected for her age and history of dyslipidemia, but no atherosclerotic plaque or stenosis was observed.

**Figure 2:**
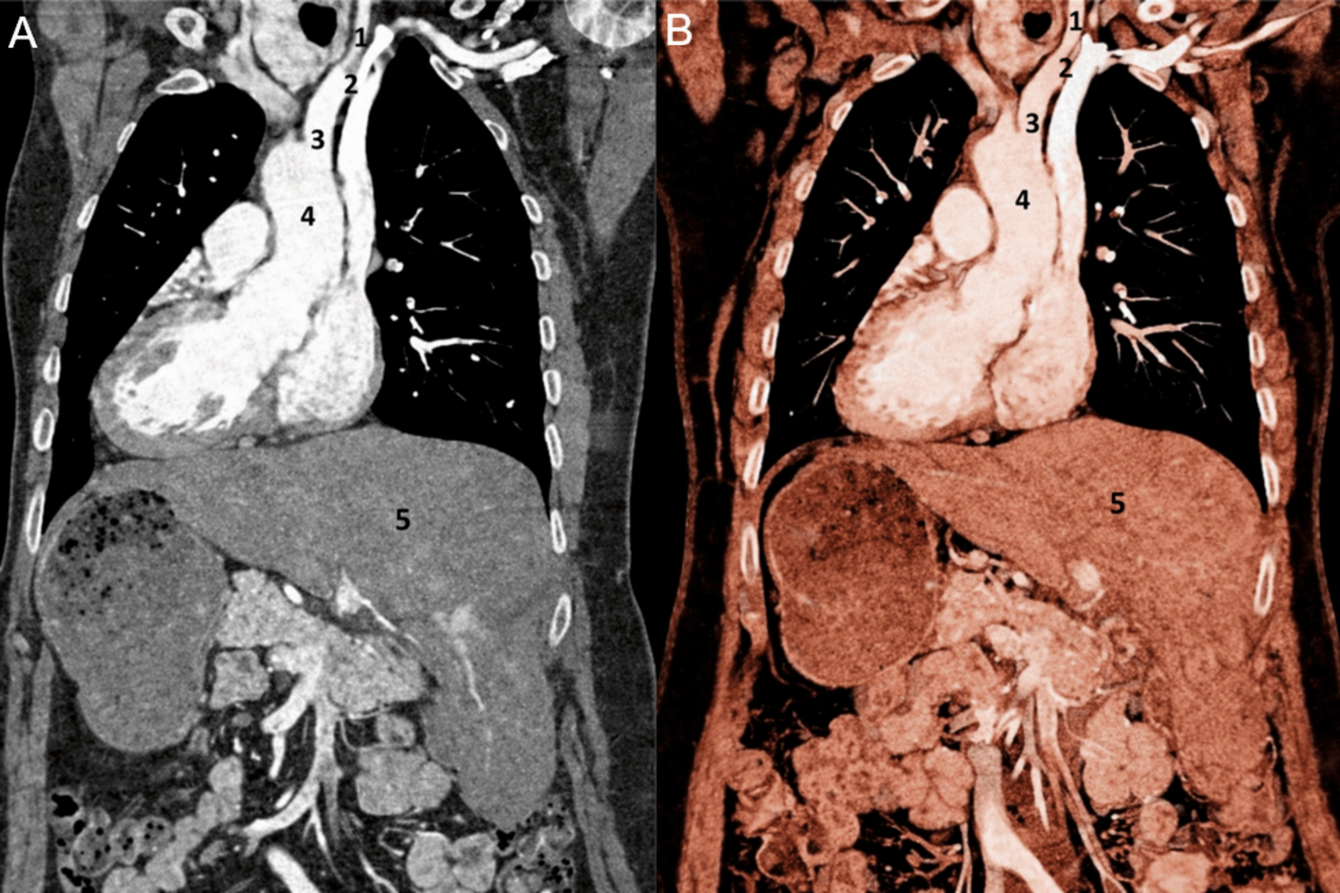
Thoracoabdominal computed tomography angiography (CTA) demonstrating the origin of the great vessels from the aortic arch and mirror-image visceral anatomy. (A) Coronal maximum intensity projection image. (B) Three-dimensional volume-rendered reconstruction. 1: left common carotid artery; 2: left subclavian artery; 3: left brachiocephalic trunk; 4: aortic arch; 5: liver

**Figure 3:**
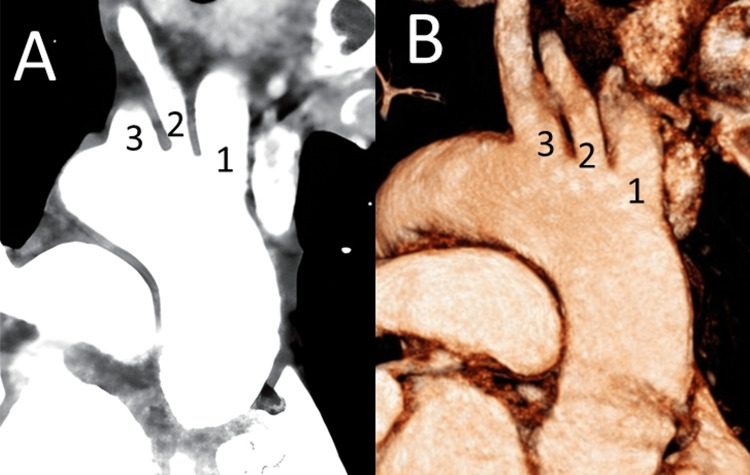
Thoracic computed tomography angiography (CTA) with focused visualization of the aortic arch and its branches, demonstrating the separate origins of the right common carotid artery (2) and right subclavian artery (3), as well as the left brachiocephalic trunk (1) arising from the aortic arch. (A) Coronal maximum intensity projection. (B) Three-dimensional volume-rendered reconstruction from a right lateral perspective.

The inability to identify the brachiocephalic trunk in its expected right supraclavicular location, combined with the identification of a large arterial trunk on the left side, raised suspicion for mirror-image anatomy rather than technical failure. Consequently, a contrast-enhanced computed tomography angiography (CTA) of the thorax and abdomen was performed during the arterial phase to delineate the full extent of the vascular and visceral anatomy. It confirmed the ultrasound findings and revealed complete SIT. The CTA demonstrated a right-sided aortic arch with mirror-image branching. The first branch was a left brachiocephalic trunk giving rise to the left common carotid and left subclavian arteries, followed by the right common carotid artery and the right subclavian artery arising separately from the arch. Furthermore, the CTA demonstrated complete mirror-image transposition of the thoracic and abdominal viscera. The heart was situated in the right hemithorax with the apex pointing to the right, consistent with dextrocardia. The liver was on the left side, the spleen and the stomach on the right side. No additional congenital anomalies, such as cardiac septal defects or polysplenia or asplenia syndromes, were identified (Figures 2, 3).

**Figure 4:**
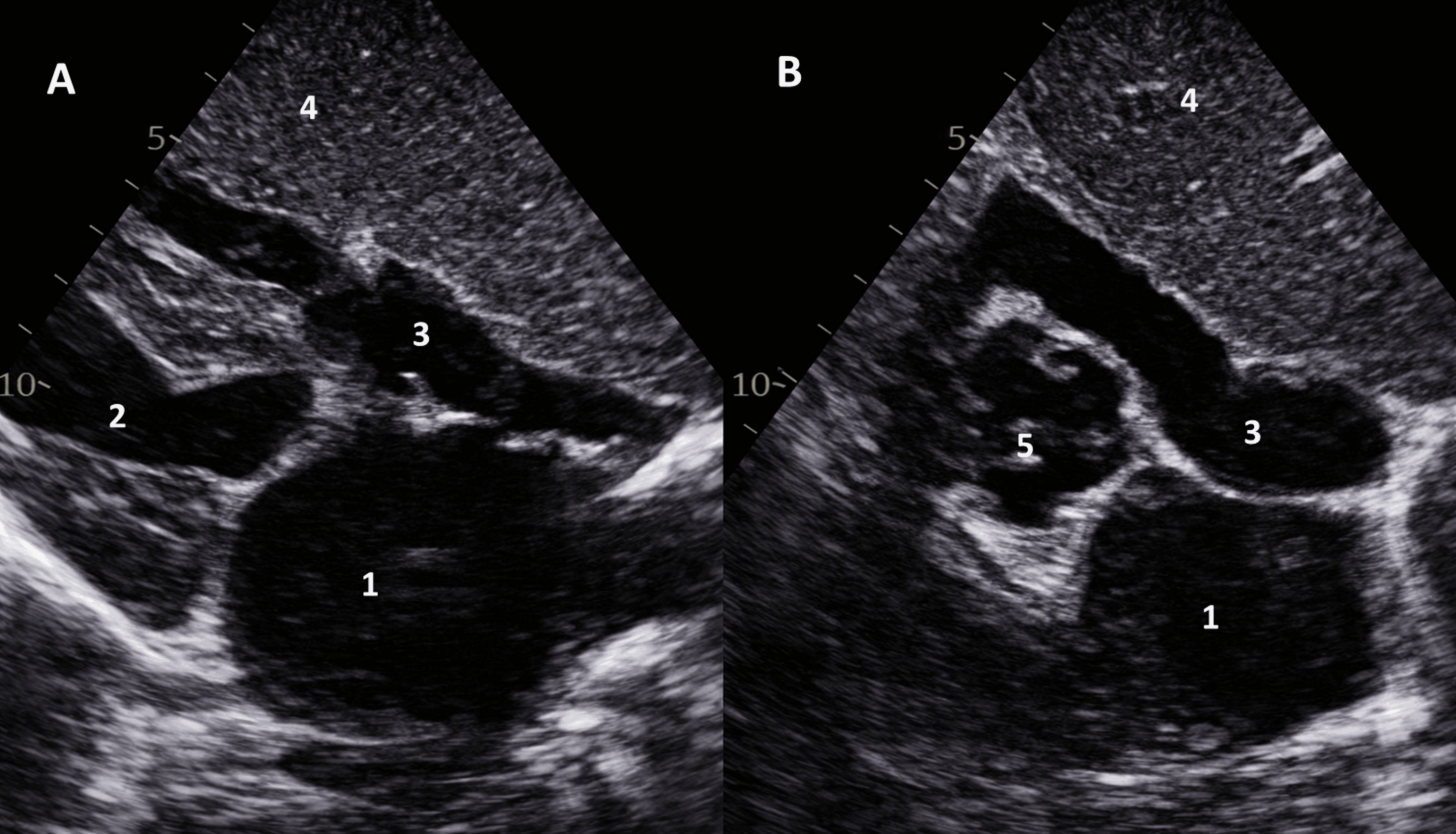
Transthoracic echocardiography (TTE) in the subcostal view demonstrating the liver on the left side. (A) Long-axis view; (B) short-axis view. 1: left atrium; 2: left ventricle; 3: right atrium; 4: liver; 5: aortic valve

Transthoracic echocardiography (TTE), using a right-sided approach, showed a left-sided liver and normal right and left ventricular function, with no valvular abnormalities (Figure 4).

The patient was informed of these findings and reassured of their benign nature, given the absence of symptoms at her age. No specific treatment or intervention was required. She was advised to inform all healthcare providers, particularly prior to surgical or interventional procedures, to ensure safe management.

## Discussion

SIT is a rare congenital malformation of normal left-right embryonic asymmetry. The left-right asymmetry is developed during the early embryonic period by ciliary flow on the primitive node, and later disturbances in this procedure can result in subsequent laterality disorders such as SIT [2]. Its exact etiology remains unidentified most of the time, but it is believed to be related to genetic mutations of the signaling cascades that define laterality [6]. About 20-25 percent of patients with primary ciliary dyskinesia have situs inversus and a condition known as Kartagener syndrome; however, the majority of patients with isolated SIT do not show signs of ciliary dysfunction [7].

In SIT, the aortic arch and its branches typically follow a mirror-image configuration rather than representing a true vascular anomaly. A normal aortic anatomy has the left aortic arch forming three branches: the brachiocephalic trunk that forms the right common carotid artery and right subclavian artery, the left common carotid artery, and the left subclavian artery [8]. In contrast, SIT is associated with a right-sided aortic arch and a mirrored brachiocephalic trunk, which splits into the left common carotid and left subclavian arteries, then right common carotid and right subclavian arteries [5]. The vascular findings in our patient are consistent with this expected mirror-image anatomy.

The key feature in this case was not a true absence of the brachiocephalic trunk, but rather its absence in the expected right-sided location during carotid duplex ultrasound. This unexpected finding prompted further evaluation and raised suspicion of mirror-image anatomy. Recognizing this distinction is important, as misinterpreting the finding as a technical failure or isolated vascular anomaly could delay the correct diagnosis.

The inability to identify the brachiocephalic trunk in its usual position on ultrasound should prompt careful reassessment. Differential considerations include technical factors such as suboptimal probe positioning, limited acoustic windows, or operator inexperience, as well as anatomical variants such as right aortic arch without situs inversus or aberrant subclavian artery. Therefore, a systematic approach and awareness of anatomical landmarks are essential during vascular ultrasound examinations. In this case, extending the examination to the contralateral side allowed identification of the mirrored vascular configuration.

Awareness of situs inversus is also clinically relevant since mirror-image anatomy could pose a problem with surgical orientation and vascular access during the cardiothoracic or interventional surgical procedure [9]. This case highlights that carotid duplex ultrasound may provide the first clue when findings do not match expected anatomy. However, ultrasound alone is insufficient to confirm the diagnosis, and further imaging, such as CTA, is required to fully evaluate both vascular and visceral anatomy.

Finally, this case illustrates that congenital conditions such as SIT can remain undiagnosed until late adulthood in the absence of symptoms. It also emphasizes the importance of maintaining a high index of suspicion when imaging findings do not align with normal anatomical expectations. Careful observation and a systematic approach during routine examinations can lead to the recognition of clinically relevant anatomical variations.

This case report has several limitations. The results might not be applicable to all SIT patients because the study is a single case study. Moreover, genetic testing was not done to eliminate other related disorders like primary ciliary dyskinesia.

## Conclusions

This case illustrates the incidental discovery of complete SIT in a 60-year-old woman during a carotid duplex ultrasound ordered to evaluate subclinical atherosclerosis. The key finding was that the expected right-sided brachiocephalic trunk was not identified, which prompted confirmatory CTA. This case highlights the crucial role of recognizing atypical vascular anatomy during standard diagnostic procedures. Significant congenital conditions can remain clinically silent and be discovered incidentally in older adults. Clinicians should remain vigilant for anatomical variations, as their identification has profound implications for patient safety in future medical and surgical interventions.
